# Pattern recognition receptor ligand-induced differentiation of human transitional B cells

**DOI:** 10.1371/journal.pone.0273810

**Published:** 2022-08-30

**Authors:** Jourdan K. P. McMillan, Patrick O’Donnell, Sandra P. Chang

**Affiliations:** 1 Department of Tropical Medicine, Medical Microbiology and Pharmacology, John A Burns School of Medicine, University of Hawaii at Manoa, Honolulu, HI, United States of America; 2 Kapiolani Medical Center for Women and Children, Hawaii Pacific Health, Honolulu, HI, United States of America; Stanford University School of Medicine, UNITED STATES

## Abstract

B cells represent a critical component of the adaptive immune response whose development and differentiation are determined by antigen-dependent and antigen-independent interactions. In this study, we explored the effects of IL-4 and pattern-recognition receptor (PRR) ligands on B cell development and differentiation by investigating their capacity to drive the in vitro maturation of human transitional B cells. In the presence of IL-4, ligands for TLR7/8, TLR9, and NOD1 were effective in driving the in vitro maturation of cord blood transitional B cells into mature, naïve B cells as measured by CD23 expression, ABCB1 transporter activation and upregulation of sIgM and sIgD. In addition, several stimulation conditions, including TLR9 ligand alone, favored an expansion of CD27^+^ IgM memory B cells. Transitional B cells stimulated with TLR7/8 ligand + IL-4 or TLR9 ligand, with or without IL-4, induced a significant subpopulation of CD23^+^CD27^+^ B cells expressing high levels of sIgM and sIgD, a minor B cell subpopulation found in human peripheral blood. These studies illustrate the heterogeneity of the B cell populations induced by cytokine and PRR ligand stimulation. A comparison of transitional and mature, naïve B cells transcriptomes to identify novel genes involved in B cell maturation revealed that mature, naïve B cells were less transcriptionally active than transitional B cells. Nevertheless, a subset of differentially expressed genes in mature, naïve B cells was identified including genes associated with the IL-4 signaling pathway, PI3K signaling in B lymphocytes, the NF-κB signaling pathway, and the TNFR superfamily. When transitional B cells were stimulated in vitro with IL-4 and PRR ligands, gene expression was found to be dependent on the nature of the stimulants, suggesting that exposure to these stimulants may alter the developmental fate of transitional B cells. The influence of IL-4 and PRR signaling on transitional B cell maturation illustrates the potential synergy that may be achieved when certain PRR ligands are incorporated as adjuvants in vaccine formulations and presented to developing B cells in the context of an inflammatory cytokine environment. These studies demonstrate the potential of the PRR ligands to drive transitional B cell differentiation in the periphery during infection or vaccination independently of antigen mediated BCR signaling.

## Introduction

Vaccination is recognized as one of the most effective approaches for infectious disease prevention. However, many new and existing vaccines require the use of immune modulators, also known as “adjuvants”, to enhance their effectiveness and durability, particularly in the very young and the elderly [1, 2]. The recognition of pattern-recognition receptor (PRR) ligands, also known as pathogen-associated molecular patterns (PAMPS), as potent immune modulators has generated a newly-defined category of immunostimulatory adjuvants that activate PRRs. Some existing vaccines contain intrinsic PAMPS, such as the Yellow Fever vaccine that activates TLR2, 7, 8, and 9 [3] while others utilize exogenous PRR ligands like MPL, a TLR4 ligand, which is adsorbed to alum in the recombinant HPV vaccine Cervarix [4, 5]. Much of the previous research investigating the cellular effects of PRR ligands has focused on their effects on dendritic cells [6–8], however B cells also may serve as direct targets of adjuvant immunomodulation and are the focus of this study.

Mechanistic studies of PRR ligands as adjuvants in the mouse model have provided important insights into the influence of PAMPS on the immune response. However, PRR mechanisms defined in the mouse model often do not translate directly to humans [9, 10] because of species-specific differences in PRR expression and responsiveness. In addition, the spectrum of PRR receptors has increased in recent years and, along with Toll-like receptors (TLRs), now includes Nod-like receptors (NLRs) and C-type lectin receptors (CLRs), among others [11]. While the effects of a few PRR ligands on human B cells have been explored [12, 13], knowledge of their influence on the differentiation and activation of B cell subsets important for immunoglobulin production and B cell memory, critical features of effective antibody-mediated immunity, is limited.

Transitional B cells are immature B cells expressing a functional B cell receptor that have recently migrated from the bone marrow to the periphery following negative selection [14, 15] and are important intermediates in the development of mature B cells [16]. In secondary lymphoid organs, transitional B cells give rise to mature B cell populations, including follicular (FO) B cells and marginal zone (MZ) B cells. FO B cells play a central role in germinal center formation in response to T cell-dependent antigens, and are responsible for the production of high-affinity, class-switched antibody and the generation of long-lived plasma cells and memory B cells [17]. MZ B cells respond to blood borne pathogens and T-independent type 2 antigens, differentiate into short-lived plasma cells, and capture and shuttle antigen from the periphery to follicular dendritic cells [18, 19].

Total B cell numbers and B cell subset frequencies vary depending on an individual’s age. B cell populations in young children differ from adults in number and proportion of immature to mature B cells. In addition to the gradual decrease in total B cell count from newborn to six years of age, there is a switch in prominent B cell populations from mostly immature/transitional B cells in early childhood to mature and memory B cells in young adults [20]. Since many vaccines are administered during childhood, it is important to examine the potential effects of immunomodulators such as PRR ligands on the maturation of the transitional B cell population prominent in newborns and young children. In addition to antigen-specific B cell receptors, transitional B cells express several PRRs that may play a role in their maturation [21]. Transitional B cells may encounter PRR ligands during infection or immunization; however, the response of human transitional B cells to these stimuli remains largely unknown. To date, the effects of a few TLR ligands on transitional B cells have been explored [22–25]. To fully understand the adjuvant potential of PRR ligands, it is important to study their ability to influence the maturation and differentiation of immature B cells into B cell populations capable of participating in the germinal center reaction and in germinal center-independent immune responses. The current study explores the effect of PRR ligands currently being investigated as vaccine adjuvants on the maturation of human transitional B cells, as demonstrated by phenotypic and gene expression changes. In addition, the transcriptomes of transitional B cells and follicular B cells are compared in an effort to identify novel differentiation antigens that distinguish these two populations and may serve as additional markers of B cell differentiation.

## Materials and methods

### B cell isolation

Cryopreserved human cord blood mononuclear cells were obtained from Hemacare (Van Nuys, CA), Lonza (Alpharetta, GA), and STEMCELL Technologies Inc. (Vancouver, BC) and used as a source of neonatal transitional B cells. Human tonsils were obtained following routine tonsillectomies from the Kapiolani Medical Center for Women and Children, Hawaii Pacific Health System (Honolulu, HI) and were used as a source of mature, FO B cells. These studies were reviewed and determined not to be human subjects research by the institutional review boards of the University of Hawaii and Hawaii Pacific Health. Freshly isolated tonsils were soaked overnight in Hank’s Buffered Saline Solution (HBSS; Thermo Fisher Scientific, Waltham, MA, USA) with 1x penicillin-streptomycin. Tonsils were minced and homogenized through a 40um cell strainer. Tonsil mononuclear cells were isolated by density-gradient centrifugation using Ficoll-Hypaque (GE Healthcare, Chicago, Il, USA). Mononuclear cells were collected at the interphase and washed in 1x HBSS. All cells were resuspended in HBSS with 2% fetal bovine serum for downstream fluorescence-activated cell sorting (FACS) and analysis.

### Antibodies and reagents

The following fluorochrome-conjugated anti-human antibodies and fluorescent dyes were used for flow cytometry analyses: CD19-BV605, CD3-BV421, and CD23-BV421 (BD Biosciences, Franklin Lakes, NJ, USA); IgM-APC (Biolegend, San Diego, CA, USA); and CD19-eVolve 605, CD38-PE-eFluor 610, CD24-APC-eFluor 780, CD27-APC, CD21-PerCP-eFluor710, IgD-PE, CD5-APC, 7AAD, Fixable Viability Dye eFluor 506, Propidium Iodide, Rhodamine 123 (R123) (Thermo Fisher Scientific). Resiquimod (R848) and glucopyranosyl lipid A (GLA) were obtained from the Infectious Disease Research Institute (Seattle, WA). C12-iE-DAP (iE-DAP), polyinosinic-polycytidylic (Poly I:C), trehalose-6,6-dibehenate (TDB), and CpG ODN 2006 (CpG) were purchased from InvivoGen (San Diego, CA).

### Transitional B cell culture conditions

FACS-sorted transitional B cells (CD3^-^ CD19^+^ Rhodamine123^hi^ CD24^+^ CD38^+^) were seeded in a 96-well U-bottom plate at 5 x 10^5^ cells/mL in RPMI containing 10% FBS and 1x penicillin-streptomycin (Thermo Fisher Scientific). Transitional B cells were cultured in medium supplemented with IL-4 (100ng/mL; PeproTech, Rocky Hill, NJ, USA), R848 (TLR7/8 ligand; 5ng/uL), GLA (TLR4 ligand; 2ng/uL), CpG ODN 2006 (TLR9 ligand; 0.25uM), iE-DAP (NOD1 ligand; 2.22ng/uL), TDB (Mincle ligand; 20ng/uL), or Poly(I:C) (TLR3 ligand; 20ng/uL). After 2 and 4 days, transitional B cell cultures were analyzed for mature B cell surface markers (CD19^+^ R123^lo^ CD23^+^) by flow cytometry. Each experiment was repeated on different samples at least 4 times. Representative data and compiled results of replicate experiments are shown.

### Flow cytometric analysis

Flow cytometry sorting and analyses were performed on a FACSAria instrument (BD Biosciences, San Jose, CA) and an Attune NxT instrument (Thermo Fisher Scientific), respectively, at the Cellular and Molecular Immunology Core Facility at the John A. Burns School of Medicine, University of Hawaii at Manoa. Cells were stained with the appropriate antibodies and incubated in the dark at 4°C for 30 minutes. Cells were then washed twice and resuspended in 1x HBSS supplemented with 2% FBS for sorting and analyses. Analyses were performed using FlowJo data analysis software (FlowJo, Ashland, OR).

### RNA sequencing

Cord blood transitional B cells (CD19^+^ CD3^-^ CD24^+^ CD38^+^ R123^hi^) and tonsil follicular B cells (CD19^+^ CD3^-^ CD23^+^ IgD^+^) were sorted and total RNA was extracted using the RNeasy Mini kit (Qiagen, Germantown, MD). Total RNA was submitted to the Genomics Shared Resource at the University of Hawaii Cancer Center for library preparation and sequencing. Total RNA was quantified and RNA quality checked using the Bioanalyzer RNA 2100 Pico instrument (Agilent, Santa Clara, CA). Libraries were prepared with AmpliSeq for Illumina Transcriptome Human Gene Expression Panel (Illumina, San Diego, CA). Sequencing was performed on a NextSeq 500 instrument (Illumina, San Diego, CA). Raw FASTQ files were transferred to the Bioinformatics core at the John A. Burns School of Medicine, University of Hawaii at Manoa for analysis. Raw reads were processed using *CutAdapt* [26] and aligned to human genome (hg38) using *STAR* [27]. Gene counts were quantified using *Partek E/M Quantification* (Partek Inc., St. Louis, MO). *DESeq2* [28] was used to analyze differential gene expression and data were visualized using R. Ingenuity Pathway Analysis (Qiagen Bioinformatics, Redwood City, CA) was used for pathway and network analysis.

### Real-time qPCR

Total RNA was reverse transcribed into cDNA using a commercial cDNA synthesis kit (Bio-Rad Laboratories, Hercules, CA, USA). For AmpliSeq confirmation and gene expression analysis following transitional B cell stimulation, cDNA was added to SYBR Green Supermix (Bio-Rad Laboratories) and FCER2, PTPN6, ADAM28, RUNX1, PRDM1, TNFRSF17 and CD27 primers (Qiagen). All real-time qPCR analyses were run on an Applied Biosystems 7500 Real-Time PCR System (Thermo Fisher Scientific). Expression fold-change was calculated using ACTB as the housekeeping gene.

### Statistical analysis

Statistical analysis was performed using GraphPad Prism 6 (GraphPad Software, San Diego, Ca). Unpaired Student’s *t* tests were employed where appropriate. Data are represented as means +/- SEM. Statistical significance was determined as follows: p > 0.05 (not significant); *p ≤ 0.05 or **p ≤ 0.01 (significant).

## Results

### Identification of transitional B cell subset from human cord blood and peripheral blood

**Fig 1:**
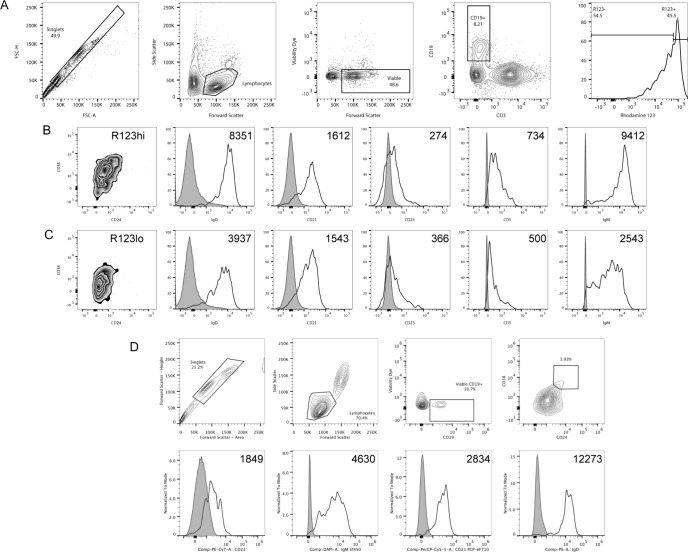
Transitional B cells from human cord blood and peripheral blood mononuclear cells. Transitional B cells were FACS-sorted based on expression of surface markers, CD19, CD24, CD38 and staining by Rhodamine 123. (A) Total cells were gated based on lymphocyte morphology, followed by gates on viable CD19^+^CD3^-^ cells, and finally Rhodamine 123 fluorescence. (B and C) Expression of sIgD, CD21, CD23, CD5, and sIgM are shown as histogram plots of median fluorescence intensity (MFI) for high (B) and low (C) Rhodamine 123 staining B cells. (D) Freshly isolated PBMCs were labeled with fluorochrome-conjugated antibodies against CD19, CD24, CD38, CD21, CD23, IgD, and IgM. Total cells were gated based on lymphocyte morphology, followed by viable CD19^+^ cells, and CD38^hi^ CD24^hi^ expression. Expression of CD23, sIgM, CD21, and sIgD by these CD38^hi^ CD24^hi^ transitional B cells are shown as MFI histogram plots (shaded histograms are isotype controls).

Transitional B cells are the recent emigrant, immature B cells from the bone marrow. Following successful migration in the periphery to secondary lymphoid organs, transitional B cells differentiate into mature, naïve B cells. To study the differentiation of human transitional B cells, we isolated these CD24^+^CD38^+^ B cells from cord blood, the richest source of human transitional B cells. Since some mature B cells were found in the CD24^+^ CD38^+^ population, rhodamine 123 (R123) retention was used as an additional marker for transitional B cells, which do not express the ABCB1 transporter responsible for efflux of R123 [29]. In contrast, mature B cells express ABCB1 and extrude this dye [30]. Approximately 50% of cord blood B cells have a transitional phenotype as defined by bright staining for R123 (Fig 1A) and high levels of CD24 and CD38 expression (Fig 1B). Consistent with the reports of others [31, 32], cord blood R123^hi^ transitional B cells also were characterized by high sIgM and sIgD expression and low or undetectable expression of CD23 (Fig 1B). While cord blood R123^lo^ cells expressed sIgM and sIgD, their expression of these markers was more heterogenous (Fig 1C) than the R123^hi^ population (Fig 1B). Moderate CD21 expression was observed in both R123^hi^ and R123^lo^ B cells (Fig 1B and 1C).

In humans, CD24^hi^ CD38^hi^ transitional B cells are also found in peripheral blood, although at a much lower frequency than in cord blood (approximately 4% of total peripheral blood CD19^+^ cells) (Fig 1D). R123^hi^ peripheral blood transitional B cells were more heterogeneous in their expression of the various cell surface markers (Fig 1D) than R123^hi^ cord blood transitional B cells (Fig 1B), making them more similar to R123^lo^ cord blood B cells (Fig 1C).

### Transitional B cells mature following IL-4 and PRR stimulation

IgD^+^CD27^-^ cord blood B cells, which may contain both transitional and mature phenotypes, and adult peripheral blood B cells are known to express TLR 1, 2, 4–10, with the highest expression of TLR 7, 9, 10 [13]. The presence of these TLRs suggests that cord blood B cells have the potential to respond to agonists of these receptors. Our first objective was to identify PRR ligands that have the ability to drive the maturation of cord blood transitional B cells. Mature B cell subsets in human secondary lymphoid organs, such as tonsils, express varying levels of surface CD21, CD23, IgD, and IgM [33, 34]. CD23 expression has been used as a marker of mature, follicular B cells [35–38] and isolated transitional B cells express CD21 with little to no CD23 (Fig 1B). Therefore, we evaluated CD23 expression as a potential marker of B cell maturation in transitional B cell cultures stimulated with various PRR ligands.

Preliminary studies of transitional B cells maintained in standard culture medium resulted in very low B cell survival, consistent with the apoptotic fate of most transitional B cells [39]. Others have reported that mouse transitional B cell survival may be enhanced by IL-4 [32]. Therefore, IL-4 was included in all transitional B cell cultures unless otherwise noted.

**Fig 2:**
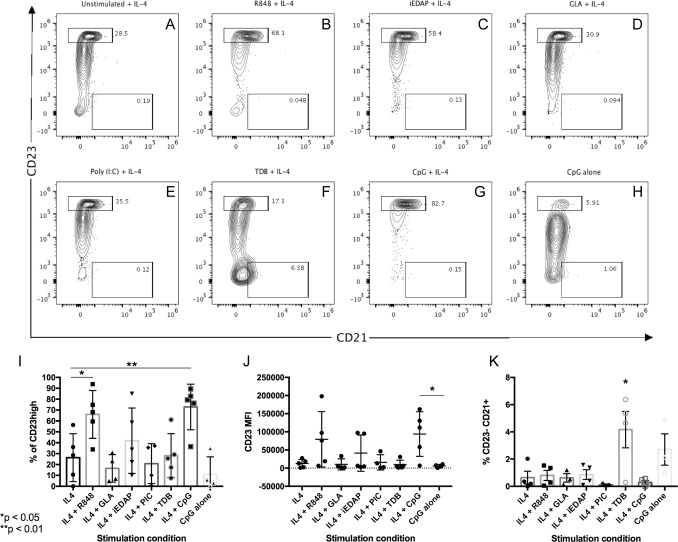
Transitional B cell differentiation into follicular-like or marginal zone-like phenotypes. FACS-sorted transitional B cells were cultured with IL-4 and individual PRR ligands at 5x10^5^ cells/mL for 2 days. Expression of CD23 and R123 exclusion were evaluated as indicators of transitional B cell maturation. R123 negative, CD23^hi^ cells (upper boxes) correspond to mature, FO-like B cells. R123 negative, CD23^-^, CD21^+^ (bottom boxes) correspond to either immature or MZ-like B cells. Purified transitional B cells were cultured with (A) IL-4 alone; (B) R848+IL-4; (C) iE-DAP+IL-4; (D) GLA+IL-4; (E) Poly (I:C)+IL-4; (F) TDB+IL-4; (G) CpG+IL-4; and (H) CpG alone. (I-J) Compiled data of five independent experiments measuring percentages of CD23^hi^ cells (I); CD23 MFI values (J), and percentage of CD23^-^CD21^+^ cells (K). Statistical significance was determined using Student’s t test for % CD23^hi^ (I), CD23 MFI (J), % CD23^-^CD21^+^ (K) values. * = p<0.05, ** = p<0.01.

To evaluate transitional B cell maturation, we stimulated highly purified cultures of CD3^-^CD19^+^ R123^hi^CD24^+^CD38^+^ transitional B cells (>95% purity) for 48- and 96-hours with PRR ligands and IL-4. Cell surface marker expression levels were the same for 48- and 96-hour cultures, therefore only 48-hour results are reported. A subpopulation of human B cells expressing variable levels of CD23 was recovered after culture of transitional B cells in the presence of IL-4 alone (Fig 2A), including some cells expressing intermediate levels of CD23, probably corresponding to maturing T2 cells, and others expressing high levels of CD23, corresponding to mature, naïve B cells [30]. IL-4 has been found to induce CD23 expression and maturation of bone marrow derived transitional B cells in mice [40] and it appears that the presence of IL-4 alone was permissive for CD23 expression by some human transitional B cells (Fig 2A).

The addition of R848, iE-DAP, and CpG to IL-4-containing cultures enhanced the maturation of transitional B cells into CD23^hi^ mature B cells, with very few CD23^-^ cells remaining at 48 h (Fig 2B, 2C and 2G). Interestingly, CpG alone (without added IL-4) preserved transitional B cell viability and induced intermediate levels of CD23 expression (Fig 2H), although relatively few cells expressed high levels of CD23 in these cultures. In cultures containing TDB plus IL-4, most B cells retained a CD23 negative, transitional phenotype (Fig 2F), with a lower percentage of CD23 high or intermediate cells as compared to IL-4 cultures. Cultures stimulated with TDB + IL-4 also contained a higher percentage of CD23^-^CD21^+^ cells than cultures receiving IL-4 alone or with other PRR ligands (Fig 2F). Poly I:C and GLA had little effect on the differentiation of transitional B cells relative to IL-4 alone (Fig 2D and 2E).

This pattern of CD23 acquisition was consistent for cord blood transitional B cell samples from five individuals (Fig 2I–2K). Transitional B cell stimulation with R848 plus IL-4 and CpG plus IL-4 resulted in the highest increase of CD23^hi^ cells (Fig 2I: R848+IL4 vs. IL4, p = 0.0148; CpG+IL4 vs. IL4, p = 0.0017) although CD23 MFIs were not significantly different from IL-4 alone cultures (Fig 2J). Stimulation of transitional B cells with CpG alone resulted in little to no increase in CD23^hi^ cells as compared to IL-4 cultures (Fig 2I) and these CD23^+^ cells displayed a significantly lower CD23 MFI than cells stimulated with CpG plus IL-4 (Fig 2J) (p = 0.0259).

The stimulation conditions used in this study generally did not detect a large CD23^-^ CD21^+^ population (Fig 2K), a phenotype associated with marginal zone B cell maturation, with one notable exception. The frequency of CD23^-^ CD21^+^ B cells was higher in cultures stimulated with TDB plus IL-4 (p = 0.0476). Cultures exposed to CpG alone as compared to IL-4 cultures tended to have higher numbers of CD23-CD21+ B cells, although this difference was not statistically significant. Other stimulation conditions resulted in an average of less than 3% of CD19^+^ B cells displaying the CD23^-^ CD21^+^ phenotype (Fig 2K). These CD23^-^ CD21^+^ cells may correspond to less mature B cells or B cells differentiating along the marginal zone B cell pathway. These results suggest that stimulation by the Mincle ligand TDB may have a distinct effect on transitional B cell maturation than ligands for TLR 7 or TLR9.

### ABCB1 identifies several mature B cell subsets within the stimulated transitional B cell population

The previous analysis focused on the expression of CD23 as a marker of B cell maturation, however since CD23 is primarily an indicator of FO B cell differentiation [41] a more general indicator of B cell maturation was of interest. The ATP-binding cassette B1 (ABCB1) transporter has been identified as a marker that discriminates mature, naïve B cells from transitional and memory B cells [29]. The acquisition of ABCB1, as reflected by the ability of cells to extrude the fluorescent dye Rhodamine 123 (R123), may be used to follow the development of transitional B cells into mature naïve B cells, including those that may not fall within the CD23^+^ population.

**Fig 3:**
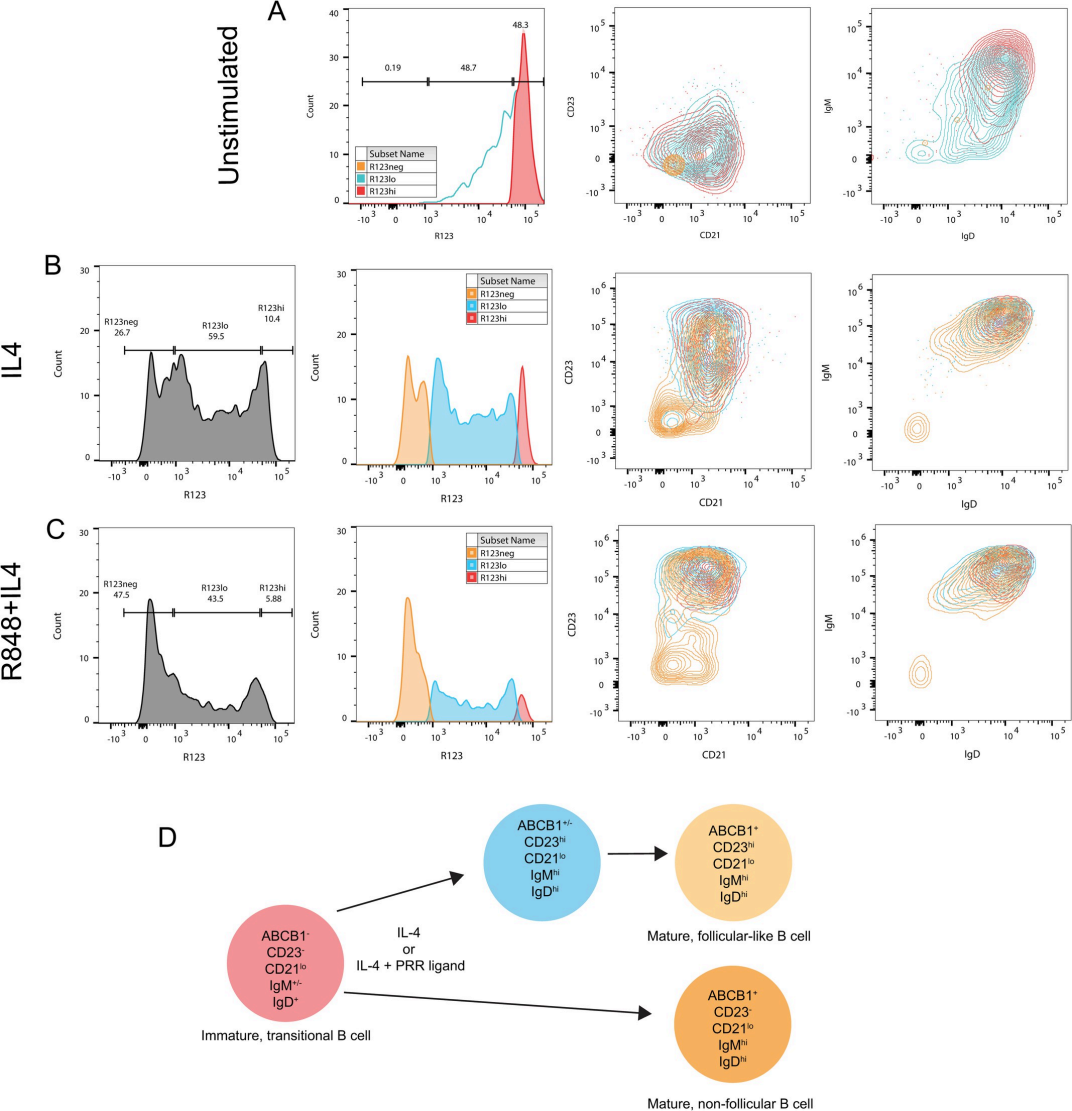
R123 expression highlights three distinct populations of immature transitional, maturing transitional, and mature B cells. (A) Baseline staining of unstimulated FACS-sorted transitional B cells with R123 (left panel), CD21 and CD23 (middle panel), and sIgD and sIgM (right panel). (B) Characteristics of transitional B cells cultured with IL-4: percentages of R123^neg^, R123^lo^, and R123^hi^ cells (panel 1); graphical distribution of R123^neg^ (orange), R123^lo^ (blue), and R123^hi^ (red) subpopulations (panel 2); CD21 and CD23 expression of R123 subpopulations (panel 3); and sIgD and sIgM expression of R123 subpopulations (panel 4). (C) Characteristics of transitional B cells cultured with IL-4+R848: percentages of R123^neg^, R123^lo^, and R123^hi^ cells (panel 1); graphical distribution of R123^neg^, R123^lo^, and R123^hi^ cells (panel 2); CD21 and CD23 expression of R123 subpopulations (panel 3); and sIgD and sIgM expression of R123 subpopulations (panel 4).(D) Model of human transitional B cell maturation stimulated by IL-4 alone or IL-4 + PRR ligand. The immature, transitional B cell possesses a characteristic phenotype as shown. Stimulation with IL-4 alone or IL-4 + PRR ligand results in the development of both CD23^+^ and CD23^-^ mature B cells that express ABCB1. The CD23^+^ population upregulates expression of CD23, sIgM, and sIgD prior to full induction of ABCB1. Expression of CD23 and ABCB1 by these B cells is markedly enhanced when they are stimulated with both IL-4 and certain PRR ligands, including R848 and CpG. The CD23^-^ population upregulates sIgM and sIgD expression and ABCB1 without inducing CD23. This population is more prominent in cells stimulated with IL-4 alone than in those stimulated with the IL-4+PRR ligand combination.

We examined the R123, CD24, and CD38 staining intensities of transitional B cells (CD19^+^ CD24^+^ CD38^+^) from cord blood mononuclear cells [30]. We observed three distinct subsets based on R123 staining: R123^hi^, R123^lo^, R123^-^ (Fig 3). Prior to culture, R123^hi^ transitional B cells were CD23^-^ CD21^lo/-^ with variable sIgM and high sIgD expression (Fig 3A, red). R123^lo^ cord blood B cells corresponding to maturing B cells that have begun to efflux R123 via the ABCB1 transporter were also observed (Fig 3A, blue). These cells were mostly CD23^-^CD21^lo/-^ sIgD^+^ with varying levels of sIgM expression, however the R123^lo^ population also contained some sIgM^-^ and sIgD^-^ cells. As expected, very few R123^-^ mature B cells (<1%) were detected in transitional B cell preparations prior to culture (Fig 3A, orange).

Culture of purified transitional B cells for two days with only IL-4, resulted in the emergence of maturing B cell populations that extruded varying amounts of R123 (Fig 3B panel 2, blue and red) as well as R123^neg^, mature B cells (Fig 3B, panel 2, orange). Fully-mature (Fig 3B, panel 3, orange) and maturing B cells (Fig 3B, panel 3, blue) have begun to express higher amounts of CD23 than the starting transitional B cells (Fig 3A, panel 2) and continue to express low amounts of CD21 (Fig 3B, panel 3). However, a minor population of R123^neg^, mature B cells were CD23^-^CD21^-^ (Fig 3B, panel 3, orange). The residual R123^hi^ cells in these cultures (Fig 3B, panel 2, red) were also positive for CD23 (Fig 3B, panel 3, red). After IL-4 culture, R123^hi^, R123^lo^, and R123^neg^ populations have increased sIgM and sIgD expression (Fig 3B, panel 4), although a minor subpopulation of R123^neg^, mature B cells were negative for both sIgM and sIgD (Fig 3B, panel 4, orange).

Cells cultured with IL-4 plus R848 displayed a higher proportion of fully mature B cells than IL-4 only cultures (Fig 3C, panel 2, orange). These mature R123^neg^ B cells included distinct subpopulations of CD23^-^, CD23^int^, and CD23^hi^ cells, with the majority of cells being CD23^hi^ (Fig 3C, panel 3). Similar to IL-4 cultures, the remaining R123^hi^ population (Fig 3C, panel 2, red) displayed a distinct phenotype from the original transitional B cell population. Interestingly, these remaining R123^hi^ cells were all CD23^hi^, sIgM^hi^, and sIgD^hi^ (Fig 3C, panels 3 and 4, red), suggesting that CD23 expression and sIgM and sIgD upregulation can occur prior to ABCB1 induction. The R123^lo^ population (Fig 3C, blue) contained a majority of sIgM^+^/sIgD^+^ cells.

Culture with IL-4 or IL-4 plus R848 consistently resulted in a mixed population of R123^neg^ B cells expressing high, intermediate, and no CD23, respectively (Fig 3B and 3C, panel 3, orange), revealing a greater heterogeneity among mature, naïve B cells than appreciated by examining CD23 expression alone (Fig 2). R848 + IL-4 induced CD23^hi^ expression in a larger proportion of cells than IL-4 alone. However, under both culture conditions, the majority of R123^neg^CD23^hi^ cells as well as R123^neg^CD23^-^ cells expressed high levels of sIgD and sIgM (Fig 3C, S1 Fig) consistent with the phenotype of mature, naïve B cells. In contrast, while the R123^neg^CD23^-^ population was mostly sIgM^hi^sIgD^hi^, minor subpopulations of sIgM^+^sIgD^lo/-^ and sIgM^-^sIgD^-^ cells were also noted (S1 Fig). While these R123^neg^ CD23^-^ B cells are presumably mature naïve B cells, some appear to have downregulated their IgM and IgD B cell receptors and may correspond to a distinct mature B cell population with regulatory activity, mature IgD^low/-^ B cells (BD_L_ cells), that develops in parallel to FO B cells [42]. Other studies have shown that TLR7 and IL-4 stimulation induce isotype class switching in activated murine B cells [43]. Since CD23 is downregulated in isotype-switched B cells [43], the small subpopulation of sIgM^-^ sIgD^-^ cells within the R123^neg^ CD23^-^ B cells alternatively may represent isotype switched B cells.

A model of transitional B cell maturation induced by stimulation with IL-4 or IL-4 and a PRR ligand is shown in Fig 3D. In this model, transitional B cells give rise to two major B cell phenotypes. The mature, follicular B cell phenotype arises from cells that upregulate CD23, sIgM, and sIgD expression prior to full induction of ABCB1. Upregulation of CD23 is strongly enhanced by dual stimulation with IL-4 and certain PRR ligands such as R848 or CpG. An alternative, non-follicular phenotype upregulates sIgM, sIgD, and ABCB1 without expressing CD23 and is enhanced by stimulation with IL-4 alone. Whether this latter population corresponds to marginal zone B cells requires further analysis.

### In vitro-generated mature B cells include IgM memory phenotypes

**Fig 4:**
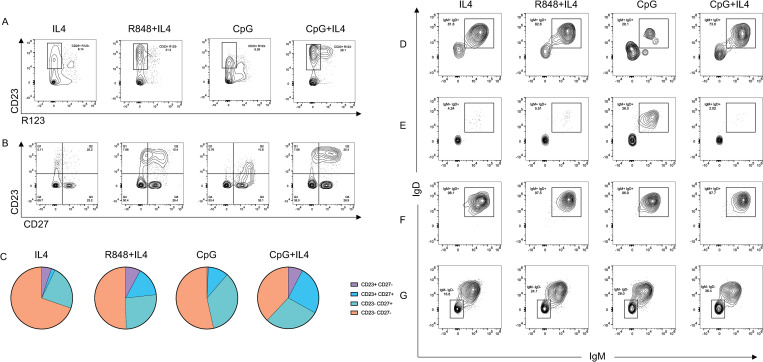
Induction of transitional B cell development into memory B cells. FACS-sorted transitional B cells were cultured with IL4, R848+IL4, CpG, or CpG+IL4 for 2 days and evaluated for expression of CD23 and the B cell memory marker, CD27. (A) Follicular B cell maturation was determined by R123 extrusion and CD23 acquisition in cultures stimulated with IL-4 (panel 1), R848+IL-4 (panel 2), CpG (panel 3), and CpG+IL-4 (panel 4). Boxed area corresponds to R123^neg^CD23^+^ mature B cells. (B) CD27 and CD23 expression of cultures stimulated with IL-4 (panel 1), R848+IL-4 (panel 2), CpG (panel 3), and CpG+IL-4 (panel 4). (C) Pie-chart display of relative amounts of cells expressing the following surface phenotypes: CD23^+^CD27^-^, purple; CD23^+^CD27^+^, blue; CD23^-^CD27^+^, green; CD23^-^CD27-, orange. Culture stimulation conditions are shown above each pie chart. (D-G) sIgM and sIgD expression of subpopulations of cells expressing different combinations of CD23 and CD27 in cultures stimulated with IL-4 (panel 1), R848+IL-4 (panel 2), CpG (panel 3), and CpG+IL-4 (panel 4).

These observations of B-cell maturation induced by IL-4 and certain PRR ligands led us to ask whether these signals, in the abscence of B cell receptor engagement with antigen, would also drive B cell memory development. CD27 expression has been associated with the development of B cell memory, including IgM memory generated by a GC-independent mechanism [44]. The following experiments evaluated whether cytokine and PRR signaling would influence induction of CD27 in stimulated B cells. As in previous experiments, human cord blood transitional B cells (CD19^+^CD38^+^CD24^+^R123^+^) were cultured with IL-4, R848+IL-4, CpG, or CpG+IL-4. As seen in Fig 4, the combination of either R848+IL-4 or CpG+IL-4 resulted in a higher percentage of CD23^+^R123^neg^ mature B cells than IL-4 or CpG alone (Fig 4A, boxed region), consistent with our previous observations. When R123^neg^ B cells were evaluated for CD23 and CD27 expression (Fig 4B and 4C), distinct patterns were observed for B cells stimulated under varying conditions. Culture with IL-4 alone resulted in a majority of CD23^-^CD27^-^ B cells (69%), a minority of CD23^-^CD27^+^ B cells (23%), and a few B cells that were CD23^+^CD27^-^ or CD23^+^CD27^+^. In contrast, cultures stimulated with CpG alone displayed a marked increase in cells that were CD23^-^CD27^+^ and CD23^+^CD27^+^, with very few cells in the CD23^+^CD27^-^ category. Cultures stimulated with R848 + IL-4 or CpG+IL-4 displayed very similar profiles to each other (Fig 4B and 4C), with an overall reduction in the percentage of CD23^-^CD27^-^ B cells and expansion of CD23^+^CD27^+^ and CD23^+^CD27^-^ populations. CpG+IL-4 cultures displayed the greatest expansion of the CD23^+^CD27^+^ population.

We then compared surface IgM and IgD expression of these various subpopulations (Fig 4D–4G). Most CD23^+^CD27^-^ B cells in all cultures, except those stimulated with CpG, expressed high levels of both sIgM and sIgD, a characteristic of mature, naïve B cells (Fig 4D). In contrast, among CD23^-^CD27^+^ B cells only cultures stimulated with CpG displayed high sIgM and sIgD expression (Fig 4E) with the majority of these cells in other cultures remaining negative for sIgM and sIgD. The CD23^+^CD27^+^ population were uniformly sIgM^hi^ sIgD^hi^, regardless of culture stimulation conditions (Fig 4F). Co-expression of sIgM, sIgD, and CD27 are a defining feature of IgM memory B cells [45]. Finally, CD23^-^CD27^-^ B cells downregulated IgD and IgM following stimulation (Fig 4G), potentially indicating immunoglobulin class switching among B cells that became sIgM^-^sIgD^-^ (Fig 4G, boxed area). Our results further subdivide these IgM memory-like B cells into two subpopulations which differ in CD23 expression. These results suggest that (1) expression of the IgM memory B cell marker CD27 can be induced in an antigen-independent manner by stimulation with TLR7/8 and TLR9 ligands along with IL-4, and (2) discrete B cell phenotypes are induced by these stimuli, when provided individually or in combination, and these B cells can be differentiated on the basis of CD23, CD27, sIgD, and sIgM expression.

### Comparison of transitional and follicular B cell transcriptomes

**Fig 5:**
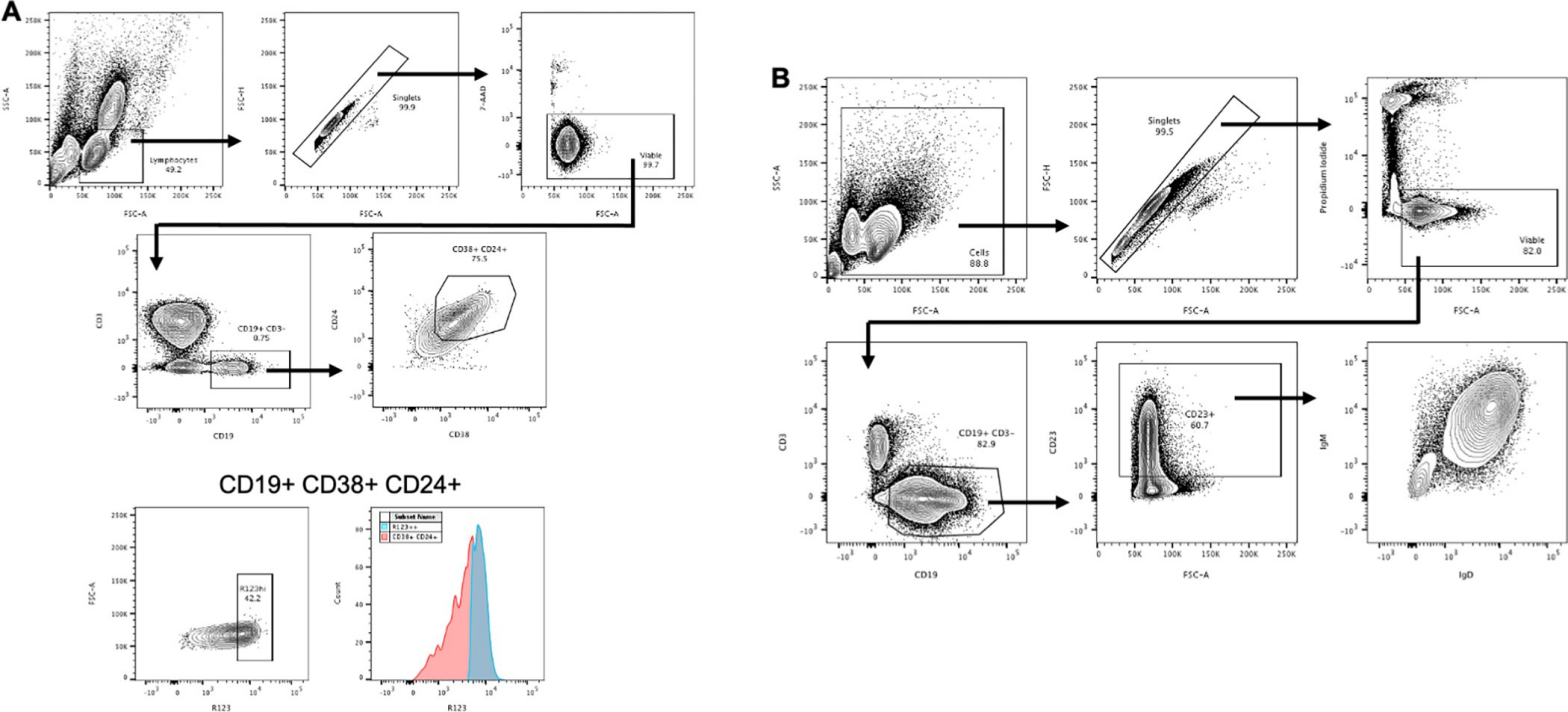
Sorting strategies for human cord blood transitional B cells and human tonsil follicular B cells. (A) Cord blood mononuclear cells were stained with R123 and chased for 3 hours. Following R123 pulse/chase, cells were stained with fluorochrome-conjugated antibodies against CD3, CD19, CD24, and CD38. Transitional B cells were sorted based on high R123 expression following the initial gating strategy. (B) Tonsil mononuclear cells were isolated from fresh tonsil tissue via mechanical homogenization and Ficoll density gradient centrifugation. Cell suspensions were stained with fluorochrome-conjugated antibodies against CD3, CD19, CD23, IgD, IgM. Follicular B cells were sorted as CD3-, CD19+, CD23+, IgD+, IgM+/-.

**Fig 6:**
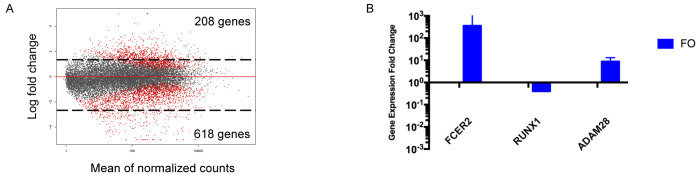
Transcriptome analysis of tonsil follicular B cells and cord blood transitional B cells. (A) Mean differences plot showing the gene expression fold change for follicular and transitional B cells. Statistically significant differences in gene expression between tonsil follicular B cells and cord blood transitional B cells are in red. (B) RT-qPCR confirmation of select genes (FCER2, RUNX1, and ADAM28) identified from the AmpliSeq analysis in purified FO and Tr B cells. Gene expression fold change is expressed as the ratio of gene expression in follicular B cells/transitional B cells.

**Fig 7:**
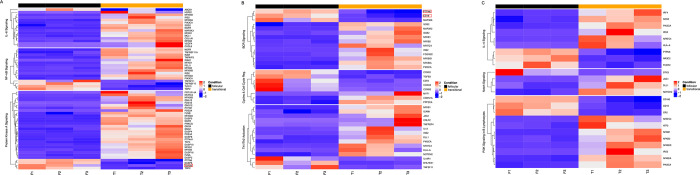
Pathway analysis of tonsil follicular B cells and cord blood transitional B cells. Heatmap display of follicular B cells (black band) and transitional B cells (yellow band) from three individual samples for each cell type. Pathways analyzed were (A) IL-6 signaling, NF-kB signaling, PKA signaling; (B) BCR signaling, Cyclins & Cell Cycle Regulation, Th1/Th2 activation; (C) IL-4 signaling, Notch signaling, PI3K signaling in B lymphocytes. Specific genes of interest are boxed in red. Heat map keys are provided on the right of each figure.

Due to the limited availability of markers associated with B cell maturation and the overlapping phenotypes among maturing and mature subsets observed in our study, our second objective was to define novel markers that may be used to distinguish transitional and mature, follicular B cell populations. These markers could be used to evaluate the maturation stage of transitional B cells activated by different stimuli. Previous transcriptional analysis of murine transitional and follicular B cell populations indicated that each have distinct gene expression profiles [46, 47]. However, limited transcriptomic data are available for isolated human B cell subsets [48]. We performed AmpliSeq analysis on purified transitional (Tr) B cells from three individual human cord blood samples and mature, follicular (FO) B cells from three individual human tonsils samples to characterize the transcriptomes of human Tr and FO B cells, respectively. Tr B cells (CD3^-^ CD19^+^ CD24^+^ CD38^+^ R123^hi^) (Fig 5A) and naïve FO B cells (CD3^-^ CD19^+^ CD23^+^ IgD^+^) (Fig 5B) were isolated by flow cytometry to >99% purity. 20,000 unique human transcripts were queried and compared between Tr and FO B cell subsets. RNASeq analysis indicated that 208 transcripts were upregulated, while 618 transcripts were downregulated in the FO B cell subset compared to the Tr B cell subset (Fig 6A). The top 20 up and down regulated genes in human tonsil FO B cells compared with human cord blood Tr B cells are shown in Table 1. Transcription of a few genes differentially expressed in FO and Tr B cells in RNASeq (Figs 6 and 7) were confirmed by real-time qPCR (Fig 6B). One of these, FCER2, encodes for CD23, an established marker of FO B cell differentiation.

**Table 1:** The top 20 upregulated and downregulated genes in tonsil follicular B cells as compared to cord blood transitional B cells.

Top 20 upregulated genes in tonsil follicular B cells				
GENE SYMBOL	DESCRIPTION	log2FoldChange	Pvalue	Padj
AC022182.2	NON-CODING RNA	5.218517081	9.25E-25	1.51E-22
AP002004.1	DYNEIN, AXONEMAL, HEAVY CHAIN 2 PSEUDOGENE	4.964714156	6.69E-11	2.09E-09
DHCR24	CHOLESTEROL BIOSYNTHESIS	4.194323615	2.22E-10	6.23E-09
RAB33A	Rab FAMILY (GTPASE SUPERFAMILY); POSSIBLE VESICLE TRANSPORT	4.178536257	2.41E-17	1.95E-15
GBP1	GUANYLATE BINDING PROTEIN; INDUCED BY IFN	4.096794862	1.12E-20	1.25E-18
LINC00643	LONG INTERGENIC NON-PROTEIN CODING RNA 643	3.977274157	1.07E-12	4.54E-11
CXorf57	RPA1 RELATED SINGLE STRANDED DNA BINDING PROTEIN, X-LINKED	3.645561721	5.99E-10	1.56E-08
MATK	TYROSINE KINASE; INHIBITOR OF SRC FAMILY KINASES	3.608789998	1.61E-29	4.10E-27
CR2	CD21; COMPLEMENT RECEPTOR 2	3.539663418	3.38E-26	6.28E-24
RTP4	RECEPTOR TRANSPORTER PROTEIN 4	3.528108116	6.47E-36	2.84E-33
FMNL3	FORMIN HOMOLOGY DOMAIN; UBIQUITOUS IN LN	3.506524256	1.30E-57	3.43E-54
CHI3L2	CHITINASE 3 LIKE 2	3.432957116	6.74E-12	2.42E-10
HJURP	HOLLIDAY JUNCTION RECOGNITION PROTEIN	3.43167608	7.68E-13	3.39E-11
TTC39B	TETRATRICOPEPTIDE REPEAT DOMAIN 39B	3.418846333	5.21E-12	1.90E-10
CCND1	CYCLIN D1	3.314808877	2.91E-16	2.10E-14
NR3C2	NUCLEAR RECEPTOR SUBFAMILY 3 GROUP C MEMBER 2	3.262200262	8.82E-11	2.74E-09
IVD	ISOVALERYL-COA-DEHYDROGENASE	3.26111869	1.99E-37	1.01E-34
LIMA1	LIM DOMAIN AND ACTIN BINDING 1	3.18135286	5.98E-14	3.08E-12
ADAM28	ADAM METALLOPEPTIDASE DOMAIN 28; NOTCH CELL-CELL INTERACTIONS	3.173104951	3.69E-21	4.38E-19
NT5E	5’-NUCLEOTIDASE ECTO; DETERMINANT OF LYMPHOCYTE DIFFERENTIATION	3.151461426	9.45E-11	2.91E-09
Top 20 upregulated genes in cord blood transitional B cells				
NR4A3	NUCLEAR RECEPTOR SUBFAMILY 4 GROUP A MEMBER 3; TRANSCRIPTIONAL ACTIVATOR	-8.088718444	9.24E-133	1.46E-128
LMNA	LAMIN A/C	-7.708745036	1.19E-60	6.27E-57
FOSL2	FOS LIKE 2, AP-1 TRANSCRIPTION FACTOR SUBUNIT; REGULATORS OF DIFFERENTIATION	-6.839376436	2.30E-28	4.91E-26
HRK	HARAKIRI, BCL2 INTERACTING PROTEIN	-6.198552912	4.09E-65	3.23E-61
GABARAPL1	GABA TYPE A RECEPTOR ASSOCIATED PROTEIN LIKE 1	-6.113576104	1.79E-49	2.17E-46
NPTX1	NEURONAL PENTRAXIN 1	-5.899527557	1.45E-15	9.56E-14
NR4A1	NUCLEAR RECEPTOR SUBFAMILY 4 GROUP A MEMBER 1	-5.6199481	4.03E-43	3.18E-40
BAIAP3	BAI1 ASSOCIATED PROTEIN 3	-5.571194202	1.21E-34	4.44E-32
CSF1	COLONY STIMULATING FACTOR 1	-5.49456572	7.18E-40	4.73E-37
PDE4A	PHOSPHODIESTERASE 4A	-5.331981408	4.21E-20	4.46E-18
DUSP8	DUAL SPECIFICITY PHOSPHATASE 8; NEGATIVELY REGULATED MAP KINASE SUPERFAMILY	-5.24340287	4.03E-35	1.55E-32
NR4A2	NUCLEAR RECEPTOR SUBFAMILY 4 GROUP A MEMBER 2	-5.098172728	1.35E-34	4.85E-32
YPEL4	YIPPEE LIKE 4	-4.836815941	3.21E-12	1.22E-10
GALNT9	POLYPEPTIDE N-ACETYLGALACTOSAMINYLTRANSFERASE 9	-4.738199381	2.20E-11	7.47E-10
LDLRAD4	LOW DENSITY LIPOPROTEIN RECEPTOR CLASS A DOMAIN CONTAINING 4	-4.716950472	6.73E-25	1.13E-22
TP53INP2	TUMOR PROTEIN P53 INDUCIBLE NUCLEAR PROTEIN 2; TRANSCRIPTIONAL ACTIVATOR	-4.683664939	1.33E-30	3.68E-28
MMP7	MATRIX METALLOPEPTIDASE 7	-4.67389253	9.34E-19	8.78E-17
AL023775.1	INSULIN LIKE GROWTH FACTOR 2 mRNA BINDING PROTEIN 3 PSEUDOGENE	-4.645116462	1.05E-21	1.27E-19
ODF3L1	OUTER DENSE FIBER OF SPERM TAILS 3 LIKE 1	-4.585112285	3.15E-11	1.04E-09
COL1A1	COLLAGEN TYPE I ALPHA 1 CHAIN	-4.556490431	5.06E-23	7.01E-21

The most highly expressed genes in FO B cells included genes associated with cell cycle regulation (CCND1, HJURP), cytoskeletal organization and cell migration (FMNL3, LIMA1), cholesterol biosynthesis and metabolism (DHCR24, TTC39B), nucleotide metabolism (NT5E/CD73), and receptor and vesicle transport (RAB33A, NR3C2) (Table 1). Among the highly upregulated genes for FO B cells as compared to Tr B cells were two long intergenic non-coding RNA (lincRNA) genes, AC022182.2 (also known as ENSG00000254802) and LINC00643 (Table 1). LincRNAs are a subset of long non-coding RNAs (lncRNAs). Expression of several lncRNAs have been reported during B-cell development and maturation [49], and it has been noted that more lncRNAs were differentially expressed between B cell subpopulations than protein-encoding genes [50], making these potential markers for B cell developmental subsets. Among the genes highly upregulated in Tr B cells were four transcription factors (NR4A3, FOSL2, NR4A2, and TP531NP2), two genes that regulate apoptosis (HRK, NR4A1), genes associated with the extracellular matrix (MMP7, COL1A1), and genes involved in the response to extracellular signals (PDE4A, BSIAP3, LDLRAD4) (Table 1).

Pathway analysis revealed an overall downregulation of FO B cells in comparison to Tr B cells with respect to transcription of genes associated with BCR signaling, PKA signaling, IL-6 signaling, Th1/Th2 activation, and cyclins & cell cycle regulation (Fig 7A–7C). Since FO B cells are resting, naïve B cells that have not yet been activated by antigen nor cognate signals from T cells, they may not be as transcriptionally active as the less mature, Tr B cell population. Nevertheless, other genes corresponding to pathways more highly represented in the FO B cell population included TGFB3 involved in Protein Kinase A signaling (Fig 7A) and protein tyrosine phosphatase non-receptor type 6 (PTPN6) and CD19 involved in BCR signaling (Fig 7B).

**Fig 8:**
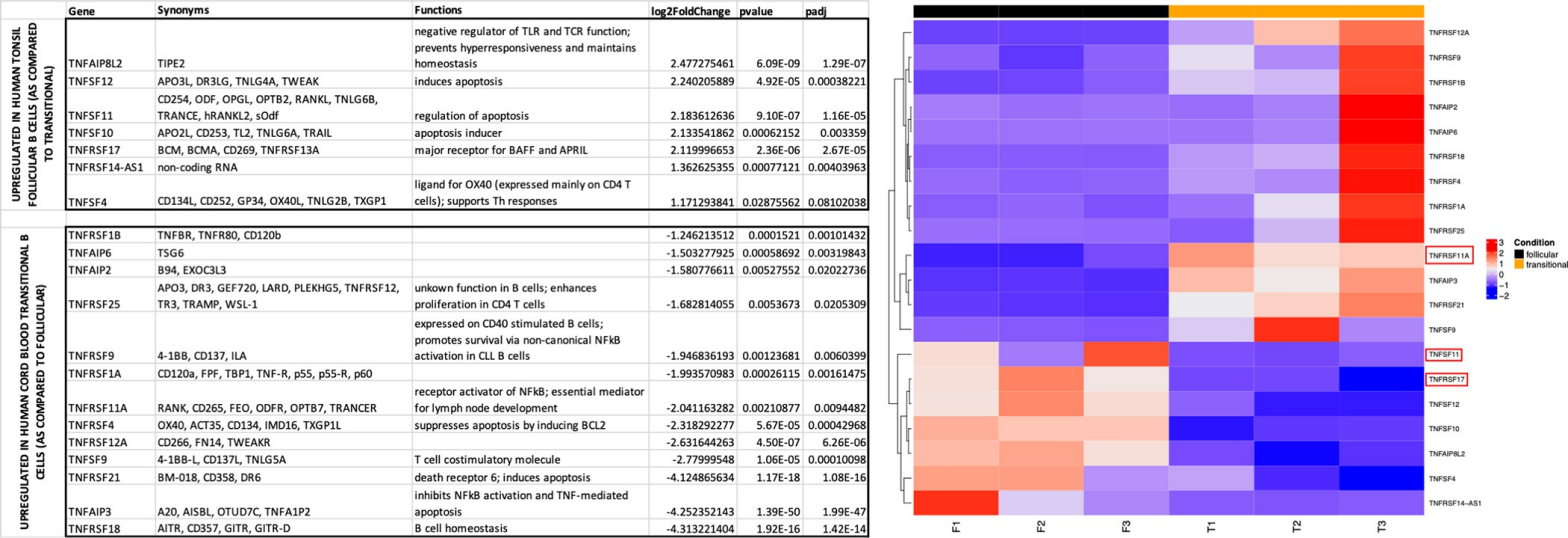
TNFR superfamily transcriptome analysis of tonsil follicular B cells and cord blood transitional B cells. Listed are genes upregulated in follicular B cells (top) and in transitional B cells (bottom). A heatmap display of follicular B cell (black band) and transitional B cell (yellow band) populations from three individuals for each cell type is shown on the right.

B cell maturation and function are highly regulated by members of the TNFR (tumor necrosis factor receptor) superfamily [51]. Our transcriptome analysis revealed differential expression of many TNFR superfamily genes (Fig 8). Expression of certain TNFR superfamily genes varied between samples. Of note, several TNFR superfamily genes were upregulated in sample T3. For this analysis, only genes that were consistently up- or down-regulated for all three samples were evaluated. TNFRSF17, encoding the B cell maturation antigen (BCMA), was one of the genes consistently upregulated in FO B cells. Genes encoding RANK (TNFRSF11A) and its ligand RANKL (TNFSF11), both members of the TNFR superfamily, were upregulated in Tr B cells and FO B cells, respectively (Fig 8).

### IL-4, R848+IL-4, CpG + IL-4 and CpG alone induce differential gene expression in transitional B cells

**Fig 9:**
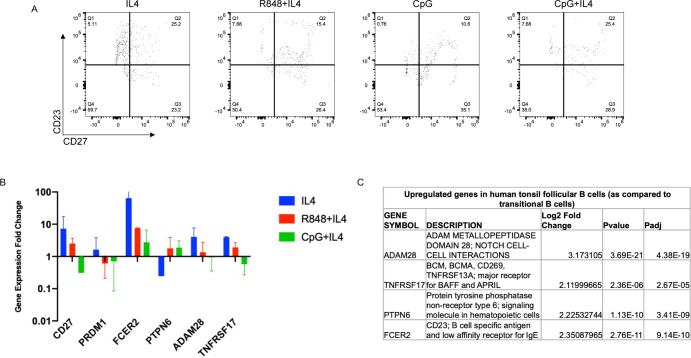
Gene expression analysis of transitional B cells stimulated with CpG+IL-4 or CpG alone. Purified cord blood transitional B cells were cultured with various stimuli, cultured cells were isolated on day 2 and cDNA was prepared for real-time qPCR. (A) CD23 and CD27 expression of cells cultured with IL-4 (panel 1), R848+IL-4 (panel 2), CpG (panel 3), and CpG+IL-4 (panel 4). (B) Gene expression fold change was calculated using CpG-stimulated cultures as the reference condition as compared to cultures stimulated with IL4 (blue), R848+IL-4 (red), and CpG+IL-4 (green). Transcripts evaluated were CD27, PRDM1, FCER2(CD23), PTPN6, ADAM28, and TNFRSF17. (C) List of upregulated genes for tonsil follicular B cells as compared to cord blood transitional B cells from transcriptome analysis.

Our AmpliSeq analysis of human cord blood transitional B cells and human tonsil follicular B cells identified several differentially expressed genes in these purified cell populations. We chose to investigate six genes as potential markers of FO B cell differentiation that may be differentially induced in transitional B cell cultures stimulated with different PRR ligands: CD27, PRDM1, FCER2 (CD23), PTPN6, ADAM28, and TNFRSF17 (BCMA) (Fig 9). Because of the overall phenotypic differences observed between cultures stimulated with CpG alone (Fig 9A), which were relatively deficient of CD23^+^ cells as compared to cultures stimulated with IL-4, CpG+IL-4 or R848+IL-4 (Fig 3), we calculated the gene expression fold change relative to cultures stimulated with CpG alone.

FCER2 encodes for CD23, a B cell antigen which was used throughout this study as a key surface marker for follicular B cell maturation. In accordance with CD23 protein expression results obtained in this experiment as well as our previous flow cytometry data (Fig 2), we confirmed that transitional B cells stimulated with IL-4, CpG+IL-4, or R848+IL-4 express higher levels of FCER2 RNA than CpG-stimulated B cells (Fig 9B).

PTPN6/Shp-1 expression was found to be upregulated in purified human tonsil FO B cells as compared to Tr B cells (Fig 7). Upregulation of PTPN6 was observed for cultures stimulated with CpG+IL-4 or R848+IL-4, while PTPN6 transcription was downregulated in cultures stimulated with IL-4 alone (Fig 9B), suggesting that the combination of PRR+IL-4 stimulation may be required for induction of PTPN6.

CD27, ADAM28 and TNFRSF17 transcription was upregulated in cultures stimulated with IL-4 or R848+IL-4 but not in cultures stimulated with CpG+IL-4. Interestingly PRDM1, which encodes the terminal B cell differentiation gene BLIMP1, was down-regulated for cultures stimulated with either R848+IL-4 or CpG+IL-4, suggesting that these stimuli do not lead to plasma cell differentiation.

In summary, FCER2/CD23 appears to remain the most consistent indicator of in vitro transitional B cell maturation in response to IL-4 as well as IL-4+PRR ligands. PTPN6 may be an indicator of PRR ligand-mediated maturation while CD27, TNFRSF17 and ADAM28 may be selectively upregulated by IL-4 and IL-4+R848 as compared to IL-4+CpG. However, it should also be noted that a mixture of CD23 and CD27 phenotypes were obtained in cell culture, particularly in cultures stimulated with R848+IL-4 and CpG+IL-4, and it may be necessary to examine individual subpopulations of cells within these cultures to determine whether expression of these genes is associated with a particular B cell subpopulation.

## Discussion

Transitional B cells are the earliest cells in the B lymphocyte lineage that appear in peripheral blood and give rise to mature B cells that develop into immunoglobulin producing cells and memory B cells responsible for the antibody-mediated immune response. While previous studies have examined the ability of PAMPs to activate mature B cells, the effect of PAMPs on the maturation of transitional B cells has not received much attention although this B cell population is enriched in infants and young children [20]. The current study examined the effect of a variety of PAMPs, also referred to as PRR ligands, on the differentiation of human transitional B cells purified from cord blood using several B cell developmental markers. CD23 was used as a marker for mature, naïve B cells that are primarily committed to the follicular B cell lineage [35, 36]. Expression of the ATP-dependent translocase ABCB1 was employed as a more general indicator of B cell maturation since it is inactive in transitional B cells but is expressed in mature, naïve B cells [29]. Finally, CD27, a B cell costimulatory molecule associated with B cell activation and differentiation, was used as a marker for memory B cells [45].

Inclusion of IL-4 was required to maintain transitional B cell viability in culture, however exposure of transitional B cells to IL-4 also resulted in maturation of a proportion of transitional B cells. These cells expressed variable levels of CD23 and ABCB1, including some cells that had completely extruded the vital dye R123, indicating their development into mature, naïve B cells. These cells additionally expressed higher levels of sIgM and sIgD than transitional B cells, another characteristic feature of mature, naïve B cells [52]. Within this R123^neg^CD23^+^sIgM^+^sIgD^+^ population, there were B cells that were CD27^-^, the expected phenotype of mature naïve B cells. However, there was also a significant population of cells that were CD23^-^CD27^+^ and lacked sIgM and sIgD expression, suggesting that some cells had developed into either antibody-secreting or memory B cells that had switched isotype or downregulated sIgM and sIgD expression [53]. IL-4 has been shown to promote class switching in human naïve B cells stimulated with CD40L [54], and these data suggest that IL-4 may itself induce class switching. Further studies are needed to examine the isotype expression and developmental stage of this B cell subpopulation. Interestingly, only CpG-stimulated cultures contained a significant population of CD23^-^CD27^+^ cells that were largely sIgM^+^ and sIgD^+^, suggesting that TLR9 stimulation alone expands a distinct mature B cell population that may correspond to marginal zone B cells.

The addition of the TLR 7/8 ligand R848 or TLR 9 ligand CpG to B cell cultures containing IL-4 resulted in both quantitative and qualitative changes. There was an enhanced upregulation of CD23 expression by B cells, with the majority of B cells expressing high levels of CD23. In parallel, a greater proportion of B cells became R123 negative, confirming that a majority of cells had developed into mature, naïve B cells. In addition, there was an expansion of the double-positive, CD23^+^CD27^+^ B cell population, a rare phenotype in cultures containing IL-4 alone, and these cells were uniformly sIgM^+^ and sIgD^+^. This latter population resembled IgM memory B cells and their development appeared to require PRR stimulation. CD23^-^CD27^+^sIgM^-^sIgD^-^ B cells, corresponding to prototypic memory B cells, were also present in these cultures at about the same proportion seen with IL-4 alone.

CpG stimulation alone was not efficient in generating the CD23^+^CD27^-^ mature, naïve B cell population but selectively induced the development of CD27^+^ B cells that were either positive or negative for CD23. Based on flow cytometry scatterplots, while the CD23^+^CD27^+^ B cells in R848+IL-4 and CpG+IL-4 cultures appeared to arise from CD23^+^ B cells, the CD23^+^CD27^+^ B cell population in CpG cultures appeared to develop from CD27^+^ B cells. Whether the CpG-induced CD23^+^CD27^+^ B cell subpopulation arises within the B cell lineage associated with marginal zone B cell development while the CpG+IL-4-induced CD23^+^CD27^+^ B cell subpopulation develops along the follicular B cell developmental pathway is of interest and remains to be determined. However, these observations raise the possibility that IgM memory B cell populations may be induced via two distinct developmental pathways, one which is induced by PRR signaling alone and another which is induced by the combination of PRR and cytokine signaling.

Our results expand upon a previous report that TLR9 ligation can preferentially drive the differentiation of human transitional B cells into mature B cells, including IgM memory B cells [51], a capability apparently shared by only a few other PRRs including TLR7/8. B cells stimulated by PRR ligation display subtly distinct developmental features, as shown by differences in CD23 and CD27 expression in cultures incubated with CpG alone as compared to CpG+IL-4. Since our in vitro cultures were initiated with purified transitional B cells, our study demonstrates that human cord blood transitional B cells respond synergistically to stimulation with IL-4 and PRR ligands. While TLR9 stimulation alone provided a strong signal for transitional B cells to develop into CD27^+^ IgM memory B cells, co-stimulation of a cytokine receptor, such as IL-4R, broadens the phenotype of developing B cells to include both mature, naïve B cells and IgM memory B cells. The detection of a prominent subpopulation of CD23^+^CD27^+^ B cells in all cultures stimulated with PRR ligands and, in particular, cultures stimulated with CpG+IL-4 was of interest since the expression of these markers are often considered to be mutually exclusive. CD23^+^CD27^+^ B cells were rare in IL-4 cultures, suggesting that PRR stimulation may be required for their development. Others have detected a small percentage of CD23^+^CD27^+^ B cells in peripheral blood of individuals at different ages [20] and this population shows a mild tendency to increase with age. It has been suggested that CD27 and CD23 may be co-expressed in a B cell subset or may represent a transient stage of B cell maturation. Since our cultures were carried out in the absence of antigen stimulation, it is possible that BCR stimulation may drive these cells to express exclusively CD23 or CD27, depending on the antigen, cytokine environment, and PRR ligand co-stimulation.

In addition to R848 and CpG, we also screened several other PRR ligands for their ability to induce transitional B cells to express CD23. In the presence of IL-4, C12-iE-DAP stimulation of NOD1, a member of the nucleotide-binding oligomerization domain-like receptor (NLR) family of proteins, resulted in a level of CD23 expression like that seen with R848 and CpG and IL-4, suggesting that this cytosolic PRR also may be able to stimulate B cell differentiation. In contrast, ligands for TLR3 and TLR4 did not have a significant effect on human transitional B cell maturation, likely reflecting the lack of TLR3 and TLR4 expression or function in these cells. Our data are consistent with other studies suggesting that human B cells express low levels of TLR3 and TLR4 [55] and confirm this property for transitional B cells as well. While, it has been reported that IL-4 induces TLR4 expression on human peripheral B cells [56], this did not appear to be the case for cord blood transitional B cells.

While CD23 expression is generally associated with follicular B cells, another population of B cells which express CD23 along with high levels of sIgM and sIgD are marginal zone precursor cells [57, 58]. In this study, stimulated cells that acquire CD23 and have extruded R123 due to the acquisition of ABCB1 may include the previously described human splenic marginal zone precursor population [59]. However, additional analyses are needed to distinguish whether the CD23^+^ B cells generated in culture correspond to follicular B cells or marginal zone precursor cells. Interestingly, previous literature has supported the ability of follicular B cells to develop into CD23^hi^ CD21^+^ marginal zone B cells and vice versa [57], suggesting that these may not represent discrete lineages.

The Mincle ligand TDB plus IL-4 inhibited the expression of CD23 of cultured B cells while slightly increasing expression of CD21 in the CD23^-^ population, suggesting an altered pattern of B cell maturation induced by this PRR. Mincle is present on peripheral B cells [60], but its expression in transitional B cells has not been previously investigated. The distinct response of transitional B cells to TDB suggests that differentiation induced by Mincle may involve a signaling or developmental pathway distinct from B cells stimulated with TLR7, TLR9 or NOD1. Mincle and other C-type lectin receptors signal through FcRγ/Syk and have been shown to induce gene expression independent of other PRR signaling pathways [61].

Our studies relied on the use of available cell surface and enzyme markers to monitor B cell development in vitro. In order to enhance our understanding of B cell maturation and development, we sought to identify additional B cell markers to track the differentiation of transitional B cells into mature, naïve B cells. To do this, we compared the transcriptomic profiles of human cord blood transitional B cells and tonsil-derived, mature naïve B cells, also referred to as follicular B cells. Pathway analysis found that the mature, naïve B cell population was transcriptionally quiescent relative to transitional B cells, with downregulation of select genes involved in BCR signaling, PKA signaling, IL-6 signaling, and cyclins and cell cycle regulation. Upregulated genes in mature, naïve B cells included PTPN6 (also known as SHP-1), CD19, and TGFB3.

Members of the TNFR superfamily also were differentially expressed in transitional and mature, naïve FO B cells. TNFRSF17, encoding the B cell maturation antigen (BCMA), was consistently upregulated in FO B cells. BCMA is a receptor that binds both BAFF and APRIL, key cytokines involved in normal B cell development. While previously literature indicates that BCMA is not required for normal B cell maturation [62], BCMA expression may contribute to FO B cell development and activation. Our analysis also demonstrated an upregulation of RANKL transcripts on FO B cells and a corresponding upregulation of RANK transcripts on cord blood Tr B cells, both members of the TNFR superfamily. The RANKL/RANK (Receptor activator of NF-kB) signaling pathway plays a key role in bone homeostasis and lymphoid tissue development [63]. Membrane-bound and soluble RANKL (TNFSF12) is produced by several lymphoid cells including activated B cells and has been shown to drive osteoclast maturation. RANK (TNFRSF11A) was originally described on dendritic cells, but has since been described on B cells [63]. Additionally, mouse studies have shown that RANK-deficiency leads to abnormal B cell development and decreased mature B cell counts in the periphery. As a recirculating B cell subset, RANKL production by FO B cells may support normal bone homeostasis. Transcription of RANK by transitional B cells is consistent with the notion that B cells require RANK for complete maturation and migration in the periphery [64].

Several other genes appeared to be selectively expressed in transitional B cells as compared to mature, follicular B cells. Transitional B cells contained a larger number of differentially expressed genes, most likely linked to their functional immaturity and their active migration to secondary lymphoid tissues. Harakiri (HRK) has been previously identified as an apoptosis regulatory gene whose product interacts with Bcl-2 to induce cell death [65]. We observed approximately 6-fold higher HRK expression in transitional B cells than in follicular B cells. HRK expression may be related to the predilection of transitional B cells to undergo apoptosis in the absence of appropriate survival signals. Comparisons between peripheral CD24^hi^ CD38^hi^ transitional B cells, maturing CD24^+^ CD38^+^, and fully mature CD24^+^ CD38^-^ B cells, have shown that HRK gene expression was highly upregulated [66], consistent with the current study. Additionally, the current study found that FOSL2 expression was preferentially elevated in the transitional B cell population. FOSL2 is a subunit of the AP-1 transcription factor, which regulates B cell proliferation and differentiation via genes essential for early B cell development including Foxo1, Irf4, and Aiolos [67]. Previous literature has implicated Aiolos as an important gene in the follicular or marginal zone B cell fate decision [68]. The elevated level of FOSL2 in transitional B cells aligns with the role of these cells as precursors of the mature follicular or marginal zone B cell subsets.

Following transitional B cell migration to secondary lymphoid organs, a fraction of these cells differentiates into mature, naïve B cells. Transcriptome analysis of tonsil B cells expressing the mature, naïve B cell phenotype identified several genes that may be important in the maturation and maintenance of this population. The CD19 gene encodes for a transmembrane glycoprotein expressed on all B cells from pro-B to terminal differentiation into plasma cells. CD19 is a critical molecule responsible for proper BCR signaling, adaptor protein recruitment, and BCR-independent signaling via the CD19/CD21 complex [69]. We observed higher CD19 expression on tonsil FO B cells as compared to cord blood Tr B cells. Previous work in the mouse model has demonstrated that CD19 is necessary for B cell survival and maintenance of the peripheral B cell pool [70]. Interestingly, B cell-specific CD19 deficiencies disrupt follicular and marginal zone populations, while the transitional B cell population remains intact. The increased CD19 expression detected in human tonsil B cells is consistent with the important role of CD19 in mature B cells.

Elevated expression of ADAM28 in the follicular B cell population was surprising as previous literature has linked ADAM28 expression to the murine MZ precursor population [58]. However, others have hypothesized that the follicular B cell population may be able to differentiate into marginal zone B cells [41]. Therefore, it is plausible that ADAM28 expression on CD23^+^ B cells denotes a subpopulation capable of marginal zone B cell differentiation. Our detection of two CD23^+^CD27^+^ IgM memory B cell populations, one derived from CD23^+^ B cells and the other from CD27^+^ B cells would be consistent with this hypothesis.

PTPN6 and TNFRSF17 (BCMA), both upregulated in follicular B cells, are important genes involved in signaling and maintenance of the follicular B cell population. The PTPN6 gene encodes for Shp-1, a well characterized tyrosine phosphatase that acts to negatively regulate BCR phosphorylation activity in resting cells [71]. B cell-specific PTPN6-deficient mice have an increased B1a population and a decreased B2 population [72], indicating that PTPN6 may play an important role in the B2 cell fate decision. The observed upregulation of PTPN6/Shp-1 protein tyrosine phosphatase in human FO B cells likely aids in their differentiation and maintenance in a resting state until antigen encounter.

The TGFB3 gene encodes for TGF-B3, a pleiotropic cytokine known to suppress murine and human B cell antibody production and proliferation [73]. In addition to the role of TGF-B3 in B cell function, resting B cells secrete TGF-B3 which can expand the regulatory T cell population [74]. As FO B cells are in a resting state, it is likely that they secrete TGF-B3 until antigen encounter or activation via other external stimuli.

The transcriptome analysis of human transitional and follicular B cell subsets has allowed for the identification of several genes unique to their respective B cell subpopulations. In this study, HRK and FOSL2 were found to be transitional B cell-specific, while FCER2, PTPN6, BCMA, and ADAM28 were follicular B cell-specific. Although not identified in the transcriptome analysis, CD27 was included to evaluate expression of this memory B cell marker in stimulated cultures. Examination of the expression of these genes by cultured transitional B cells stimulated with IL-4 and IL-4+PRR ligands confirmed that FCER2, which encodes the CD23 antigen, was consistently upregulated in cultures stimulated with IL-4 or IL-4+PRR ligands. ADAM28, TNFRSF17(BCMA) and CD27 were upregulated in cultures stimulated with IL-4 with or without a TLR7 ligand but not with IL4+TLR9 ligand. Whether ADAM28 and TNFRSF17 may serve as specific markers for distinct B cell subpopulations requires further investigation.

One limitation to this study is based on our observations that the resultant B cell cultures after IL-4 and PRR ligand stimulation typically contained a mixed population of several B cell phenotypes. It may be necessary to perform single cell sequencing on isolated B cell subpopulations to clearly identify candidate genes that may serve as specific markers of B cell differentiation.

As recent bone marrow emigrants, transitional B cells are the earliest circulating B-lineage cells to encounter antigen resulting from infection or vaccination. However, the influence of other immunostimulatory molecules, such as PRR ligands, on the developing B cell population is not well-appreciated. The effect of these immunomodulators, which are included in vaccines for their immune-adjuvant activity, on transitional B cells is of particular interest because they may qualitatively alter the resulting immune response. Such an effect would be highly relevant in children who have a higher level of circulating transitional B cells than adults [75]. Previous literature has focused on the response of naïve and memory B cell responses to immune modulators like PRR ligands [76–81]. However, these vaccine adjuvant components may also modulate transitional B cell maturation and thereby affect the phenotype of B cell populations capable of participating in the adaptive immune response. Our results indicate that not only do these PRR ligands drive transitional B cells to develop into mature, naïve B cells but they also induce the generation of IgM memory B cells and the emergence of other, less-recognized B cell subpopulations.

Since it is critical that the B cell repertoire, in the context of infection or vaccination, contain clones sufficiently diverse to recognize multiple pathogen variants, PRR stimulation may serve to broaden the resulting mature B cell and memory B cell repertoires. A dual control system for transitional B cell maturation has been proposed [82] suggesting that BCR/BAFFR signaling cooperate to produce a B cell repertoire with diverse BCR affinities. Our in vitro model suggests that IL-4 and PRR signaling may behave similarly to BAFFR signaling in this dual control system by expanding the B cell repertoire in an antigen-independent manner and rescuing B cell clones that may have otherwise been eliminated by apoptosis.

Our current studies indicate that transitional and mature B cells have unique gene expression patterns and transitional B cell maturation may be influenced by several environmental cues including IL-4 and PRR ligands. We propose that these signals work synergistically to create a diverse mature B cell repertoire necessary for a robust and efficient adaptive immune response. Further studies are needed to evaluate the impact of PRR- and IL-4-dependent, antigen-independent signaling on the B cell repertoire, as well as to define whether PRR ligands as components of adjuvant formulations may affect transitional B cell maturation in vivo.

## Supporting information

S1 Fig Surface IgM and IgD expression increases independently of CD23 expression in transitional B cell maturation. Transitional B cells were cultured with IL-4 or R848+IL-4 and examined for R123 extrusion (A), CD21 and CD23 expression (B), and sIgM and sIgD expression (C). Although CD23 expression and R123 extrusion in cultures stimulated with IL-4 alone is less than in cultures stimulated with R848+IL-4, most R123^neg^ cells in both cultures express high levels of sIgM and sIgD.
(EPS)

## Abbreviations

CLR: C-type lectin receptor
CpG: CpG polynuceotide
NLR: NOD-like receptor
MZ: marginal zone
FO: follicular
GLA: glucopyranosyl lipid A
iE-DAP: C12-iE-DAP
Poly I:C: polyinosinic—polycytidylic acid
TDB: trehalose-6,6-dibehenate
